# Association of Vitamin D Pathway Genetic Variation and Thyroid Cancer

**DOI:** 10.3390/genes10080572

**Published:** 2019-07-28

**Authors:** Isabel S. Carvalho, Catarina I. Gonçalves, Joana T. Almeida, Teresa Azevedo, Teresa Martins, Fernando J. Rodrigues, Manuel C. Lemos

**Affiliations:** 1 CICS-UBI, Health Sciences Research Centre, University of Beira Interior, 6200-506 Covilhã, Portugal; 2 Serviço de Endocrinologia, Instituto Português de Oncologia de Coimbra, 3000-075 Coimbra, Portugal

**Keywords:** thyroid cancer, vitamin D, genetics, polymorphisms

## Abstract

Vitamin D is mostly known for its role in bone and calcium metabolism. However, studies have suggested that it also has inhibitory effects on tumor development and progression. Genetic variants close to genes that encode crucial enzymes for the synthesis (*DHCR7* rs12785878), metabolism (*CYP2R1* rs2060793) and degradation (*CYP24A1* rs6013897) of vitamin D have been associated with serum levels of vitamin D. The aim of this case-control study was to determine the effect of these variants in the vitamin D pathway on the susceptibility to thyroid cancer. Five hundred patients with differentiated thyroid cancer and 500 controls were genotyped for the *DHCR7* rs12785878, *CYP2R1* rs2060793, and *CYP24A1* rs6013897 variants. Genotype and allele frequencies were compared between patients and controls. The *DHCR7* rs12785878 minor allele was associated with thyroid cancer under an additive (OR 1.38, 95% CI 1.15–1.65, *p* = 0.0004) and codominant (OR 1.88, 95% CI 1.30–2.74, *p* = 0.0021) model. These findings suggest that *DHCR7* polymorphisms may be associated with an increased risk of thyroid cancer due to an effect of this gene on circulating vitamin D levels.

## 1. Introduction

Thyroid cancer is the most common endocrine cancer [1]. The etiology of thyroid cancer remains largely unknown, but individual susceptibility to thyroid cancer is likely to be influenced by the combined effect of the environment and multiple low-penetrance to moderate-penetrance genes [2,3]. Several studies have shown that serum levels of 25-hydroxyvitamin D are lower in patients with thyroid cancer [4,5]. This is in agreement with the growing body of evidence for a role of vitamin D in regulating cell proliferation and differentiation [6,7].

There is an inter-individual variability in serum levels of vitamin D that is not explained alone by dietary intake or sunlight exposure [8]. Studies have shown that a significant proportion of the variability of vitamin D levels is due to genetic and epigenetic factors that affect several steps along the vitamin D pathway [9]. In particular, two large genome-wide association studies (GWAS), performed on individuals of European descent, have associated serum 25-hydroxyvitamin D levels with Single Nucleotide Polymorphisms (SNPs) close to genes involved in the synthesis (7-dehydrocholesterol reductase, *DHCR7*), hydroxylation (cytochrome P450 subfamily IIR polypeptide 1, *CYP2R1*) and degradation (cytochrome P450 family 24 subfamily A polypeptide 1, *CYP24A1*) of vitamin D [10,11].

*DHCR7* (on chromosome 11q13.4) encodes the enzyme 7-dehydrocholesterol reductase, which is the penultimate enzyme of sterol biosynthesis that converts 7-dehydrocholesterol (7-DHC) to cholesterol. Homozygous mutations in *DHCR7* cause the Smith-Lemli-Opitz syndrome, which is associated with elevated serum 7-DHC levels, low serum cholesterol levels, and multiple congenital abnormalities, but heterozygous carriers have been proposed to be protected against rickets and osteomalacia from hypovitaminosis D [11]. *CYP2R1* (on chromosome 11p15.2) encodes microsomal vitamin D 25-hydroxylase, which catalyzes the first hydroxylation reaction in the biosynthesis of vitamin D. Homozygous mutations in *CYP2R1* have been identified in cases of vitamin D-dependent rickets type 1B [10,11]. *CYP24A1* (on chromosome 20q13.2) encodes vitamin D 24-hydroxylase, which is involved in the inactivation of vitamin D metabolites [11]. Previous GWAS demonstrated that the rs12785878, rs2060793, and rs6013897 SNPs, located near the *DHCR7*, *CYP2R1*, and *CYP24A1* genes, respectively, were strongly associated with serum vitamin D levels in both discovery and replication cohorts [10,11].

The aim of this study was to determine if the *DHCR7* rs12785878, *CYP2R1* rs2060793, and *CYP24A1* rs6013897 SNPs are also associated with susceptibility to thyroid cancer.

## 2. Materials and Methods

### 2.1. Subjects

The study was designed as a retrospective case-control association study. Cases consisted of 500 Caucasian Portuguese patients with thyroid cancer (91 males and 409 females, mean age ± standard deviation (SD) = 46.1 ± 13.9 years) who attended the outpatient clinics at the Portuguese Institute of Oncology, Coimbra (Portugal). Patients were selected on the basis of histologically confirmed presence of any of the two major subtypes of differentiated thyroid carcinoma, i.e., papillary (*n* = 442) and follicular (*n* = 58) thyroid carcinoma. The control group consisted of 500 (113 males and 387 females, mean age ± SD = 35.9 ± 14.2 years) Caucasian Portuguese unrelated volunteers who were recruited among blood donors from the same geographical area. Cases and controls were the same as those used in a previous study showing an association of *FOXE1* variants with thyroid cancer [12], with some replacements due to missing DNA samples. The study was approved by the local research ethics committees (Portuguese Institute of Oncology of Coimbra, and Faculty of Health Sciences, University of Beira Interior, Ref: CE-FCS-2012-011). Written informed consent was obtained from patients and controls and all methods were performed in accordance with the relevant guidelines and regulations.

### 2.2. Genetic Studies

The *DHCR7* rs12785878, *CYP2R1* rs2060793, and *CYP24A1* rs6013897 SNPs were selected for this study since they had been demonstrated to be among those variants most strongly associated with circulating vitamin D levels in previous GWAS [10,11]. Minor allele frequencies (MAF) reported in the Genome Aggregation Database (gnomAD) [13] for Non-Finnish Europeans are 0.278 (rs12785878 allele G), 0.403 (rs2060793 allele A), and 0.197 (rs6013897 allele A), respectively.

Genomic DNA was extracted from peripheral blood leukocytes using previously described methods [14]. SNP genotyping was performed by a polymerase chain reaction (PCR) and restriction fragment analysis. PCR primers were designed using Primer 3 Plus [15] and consisted of 5′-CCCCTGCCTTAGTGTGTGTT-3′ and 5′-CCGGGGGCCTTTAGTTAGAC-3′ (for *DHCR7* rs12785878 T > G), 5′-TGGAGCTGAGATCAACTGCTAAA-3′ and 5′-GTGACACATACCTGTGGGGG-3′ (for *CYP2R1* rs2060793 G > A), 5′-GGACTCCTGGTTGGGTGATG-3′ and 5′-TCCTTGATCCAAATGTCCGCA-3′ (for *CYP24A1* rs6013897 T > A). Furthermore, for each SNP, 100 ng of genomic DNA were used in 15 μL reactions containing 0.25 μM of each primer, 1 U of Taq DNA polymerase and respective buffer (DreamTaq Green DNA polymerase, Thermo Fisher Scientific, Waltham, MA, USA), and 200 μM of each deoxynucleotide (dNTP). The PCR cycle conditions consisted of initial denaturation at 95 °C for 5 min, which was followed by 34 cycles of 95 °C for 30 s, 58 °C for 30 s, and 72 °C for 30 s, with a final extension step of 72 °C for 10 min. Amplified fragments were then digested with the appropriate restriction enzyme (New England Biolabs, Beverly, MA, USA or Thermo Fisher Scientific, Waltham, MA, USA), according to the manufacturer’s instructions and visualized after electrophoresis on a 2% or 3% agarose gel. The rs12785878 SNP was analyzed by the digestion of the 618-base pair (bp) PCR product with TaqI, which resulted in two fragments (320 bp and 298 bp) in the presence of the G allele and one uncut fragment (618 bp) in the presence of the T allele. The rs2060793 SNP was analyzed by the digestion of the 768-bp PCR product with SchI, which resulted in three fragments (379 bp, 248 bp, 141 bp) in the presence of the G allele and two fragments (627 bp and 141 bp) in the presence of the A allele. The rs6013897 SNP was analyzed by the digestion of the 516-bp PCR product with TscAI, which resulted in two fragments (414 bp and 102 bp) in the presence of the A allele and three fragments (222, 192, and 102 bp) in the presence of the T allele. Sequencing the PCR products of a representative individual for each of the nine possible genotypes validated the genotyping assay (GenomeLab TM GeXP, Genetic Analysis System, Beckman Coulter, Fullerton, CA, USA).

### 2.3. Statistical Analysis

Genotype and allele frequencies in patients and controls were determined by direct counting. Frequencies among groups were compared by unconditional logistic regression analysis to obtain odds ratios (OR), 95% confidence intervals (CI) and *p*-values, using SNPStats software (Catalan Institute of Oncology, Barcelona, Spain). [16] and adjusted for sex and age at diagnosis (<45 or ≥45 years). The best model of inheritance for the polymorphism (codominant, dominant, recessive, over-dominant, or additive) was selected using Akaike information criterion and SNPStats software [16]. Subgroup analysis was carried out to assess the effect of the three polymorphisms on cancer subtype (papillary vs. follicular thyroid carcinoma), tumor size at diagnosis (≤1 vs. >1 cm of diameter), and lymph node or distant metastasis (presence vs. absence). Statistical significance was corrected for multiple comparisons using the Bonferroni correction (*p* < 0.05, divided by the number of analyzed SNPs) and set at corrected *p* < 0.017. The Hardy–Weinberg equilibrium was assessed using the chi-squared goodness-of-fit test to compare the observed and allele-based expected genotype frequencies. Power calculation was carried out using the software Power and Sample Size Calculations (version 3.1.2, Vanderbilt University, Nashville, TN, USA) [17]. Assuming a minor allele frequency between 20% and 40%, it was estimated that the study sample was sufficient to detect ORs between 1.29 and 1.35, for overall thyroid cancer under an additive model of inheritance, with an estimated power of 0.8 and a type 1 error probability of 0.05.

## 3. Results

The *DHCR7* rs12785878, *CYP2R1* rs2060793, and *CYP24A1* rs6013897 genotype and allele frequencies observed in patients and controls are presented in Table 1, Table 2 and Table 3, respectively. Observed genotype frequencies did not deviate from the expected frequencies under the Hardy-Weinberg equilibrium.

The *DHCR7* rs12785878 minor allele (G allele) was associated with overall thyroid cancer under an additive (OR 1.38, 95% CI 1.15–1.65, *p* = 0.0004) and codominant inheritance model, in which the homozygous GG genotype had the greatest risk (OR 1.88, 95% CI 1.30–2.74) and the heterozygous TG genotype an intermediate risk (OR 1.41, 95% CI 1.07–1.86) (p = 0.002) (Table 1). This association was observed for both papillary (OR 1.35, 95% CI 1.12–1.62, p = 0.0016, additive model) and follicular (OR 1.64, 95% CI 1.11–2.42, p = 0.013, additive model) thyroid carcinoma. All associations remained significant after adjusting for sex and age.

No significant differences between patients and controls were observed for the *CYP2R1* rs2060793 (Table 2) and *CYP24A1* rs6013897 (Table 3) SNPs.

The distribution of SNPs did not differ with age at diagnosis (<45 vs. ≥45 years), gender (male vs. female), tumor subtype (papillary vs. follicular), tumor size (≤1 vs. >1 cm), or lymph node or distant metastasis (presence vs. absence). The *CYP2R1* rs2060793 minor allele (A allele) was less frequent in patients with a lymph node or distant metastasis (Table 2). Individuals with at least one A allele had a decreased risk of metastasis, under a dominant inheritance model (OR 0.61, 95% CI 0.40–0.93) (*p* = 0.022). However, this *p*-value was above the threshold of statistical significance (*p* > 0.017) determined by the Bonferroni correction for multiple comparisons.

## 4. Discussion

This case-control study revealed an increased frequency of the *DHCR7* rs12785878 minor allele (G allele) in patients with thyroid cancer. The homozygous GG genotype was associated with 1.9 times greater risk for thyroid cancer (OR 1.88, 95% CI 1.30–2.74), when compared to the homozygous TT genotype, whereas the heterozygous TG genotype conferred an intermediate risk (OR 1.41, 95% CI 1.07–1.86). These results suggest that this genetic variant, which previous GWAS have associated with lower circulating vitamin D levels [10,11], is also a risk locus for thyroid cancer in the Portuguese population.

To date, there are no functional studies of the rs12785878 polymorphism, so it remains unclear by which mechanism this polymorphism influences the serum levels of vitamin D and cancer risk. The rs12785878 polymorphism is located 8 kilobases upstream from the transcription initiation site of *DHCR7* and it is unknown if it has any effect on enzyme expression or if it has only an indirect effect through linkage disequilibrium with other functional variants in the region. In fact, rs12785878 is located in an intron of an entirely different gene (*NADSYN1*, encoding nicotinamide adenine dinucleotide (NAD) synthetase). Hence, the location of this SNP is often referred to as the *NADSYN1/DHCR7* locus [10,11]. However, it is *DHCR7* that has been assumed to be the candidate gene for vitamin D levels, as it encodes the enzyme 7-dehydrocholesterol reductase, which converts 7-dehydrocholesterol to cholesterol. Therefore, this removes the substrate from the synthetic pathway of vitamin D3 in the skin [10,11].

Our study is the first to report an association of *DHCR7* rs12785878 with any form of cancer. Other studies performed on colorectal [18,19,20], breast [20,21], prostate [20,21], ovarian [20], lung [20,22], pancreatic [20], neuroblastoma [20], and overall cancer risk [23] did not find an association with *DHCR7* rs12785878. However, the association of these cancers with vitamin D levels is more controversial than for thyroid cancer [4,5], and this might explain the lack of an association with *DHCR7*. No other study of this SNP in thyroid cancer has so far been reported.

The *CYP2R1* rs2060793 (G > A) and *CYP24A1* rs6013897 (T > A) variants were not found to be associated with thyroid cancer in our study, despite their reported association with lower vitamin D levels [10,11]. This is in agreement with Penna-Martinez et al. [24] who analyzed different SNPs within *CYP2R1* (rs12794714 and rs10741657) and *CYP24A1* (rs927650, rs2248137, rs2296241) and found no association of thyroid cancer with each individual SNP, even though certain haplotypes of *CYP24A1* were associated with a higher risk.

Clinical parameters (age at diagnosis, gender, tumor subtype, tumor size, presence of lymph node, or distant metastasis) were analyzed to assess if genetic susceptibility was limited to subgroups of patients, which has been reported for other genetic variants [12,25,26]. Our study revealed a decreased frequency of the *CYP2R1* rs2060793 (G > A) minor allele in patients with metastatic disease (OR 0.61, 95% CI 0.40–0.93), which suggests a protective effect of this SNP against more advanced disease. However, this association failed to reach statistical significance after correction for multiple comparisons. It is noteworthy that other genetic variants associated with lower serum vitamin D have been associated with protection against aggressive (but not nonaggressive) prostate cancer [21] and with improved survival after high-grade glioma [27]. Thus, the significance of our findings needs to be clarified in further investigations.

GWAS on thyroid cancer have not identified *DHCR7* or any other vitamin D-related gene, as risk loci for thyroid cancer [3]. Susceptibility SNPs identified in case-control association studies have not always been confirmed by GWAS [2]. This could indicate the absence of a real effect of some SNPs, but an alternative explanation is that the high stringent criteria applied in GWAS, designed to prevent false-positive findings, may exclude SNPs truly associated with the risk [2,3].

Our study has some limitations. First, serum vitamin D levels are not routinely obtained in the preoperative work-up of patients and, therefore, it was not possible to correlate the vitamin D levels with the genetic profile and with the disease status. However, measurements of circulating vitamin D also have limitations, which include inter-laboratory differences, seasonal fluctuations, fasting status, and other confounding effects caused by the disease or its treatment [28]. Second, there are no functional studies of the genetic variants to confirm their role in vitamin D homeostasis. Although the effect of the variants on cancer susceptibility may be through lower vitamin D levels, a vitamin D-independent mechanism of these variants on thyroid tumorigenesis has not been excluded. In particular, there is evidence that the DHCR7 enzyme regulates the switch between cholesterol and vitamin D production [29] and that cholesterol metabolism may also be implicated in cancer development [30]. Lastly, the associations found in this study may be population-specific and not replicated in other populations with different environmental exposures and genetic profiles. In contrast to the association of *DHCR7* rs12785878 with vitamin D levels in individuals of European descent, a study with Arab and South Asian individuals did not identify such an association [31]. Therefore, it would be of interest to replicate our study in other European and non-European populations.

In conclusion, our data suggest that *DHCR7* rs12785878 is a novel risk locus for thyroid cancer and that *CYP2R1* rs2060793 may confer a protective effect against disease progression. These findings may contribute to a better understanding of the role of vitamin D in the development and progression of thyroid cancer.

## Figures and Tables

**Table 1:** Analysis of *DHCR7* rs12785878 genotype and allele frequencies.

	Genotypes, *n* (%)			Alleles, *n* (%)	
	TT	TG	GG	T	G
**All**					
Controls (*n* = 500)	197 (39.4)	234 (46.8)	69 (13.8)	628 (62.8)	372 (37.2)
Patients (*n* = 500)	150 (30.0)	251 (50.2)	99 (19.8)	551(55.1)	449 (44.9)
OR (95% CI) ^§^	1.00	1.41 (1.07–1.86) *p* = 0.0021 ^(a)^	1.88 (1.30–2.74) *p* = 0.0021 ^(a)^		1.38 (1.15–1.65) *p* = 0.0004 ^(b)^
OR (95% CI) ^#^	1.00	1.35 (1.01–1.81) *p* = 0.0058 ^(a)^	1.84 (1.25–2.72) *p* = 0.0058 ^(a)^		1.36 (1.13–1.64) *p* = 0.0013 ^(b)^
**Tumor Type**					
FTC (*n* = 58)	14 (24.1)	31 (53.5)	13 (22.4)	59 (50.9)	57 (49.1)
PTC (*n* = 442)	136 (30.8)	220 (49.8)	86 (19.5)	492 (55.7)	392 (44.3)
OR (95% CI) ^§^	1.00	0.73 (0.38–1.42)	0.68 (0.31–1.52)		0.82 (0.56–1.22)
OR (95% CI) ^#^	1.00	0.76 (0.39–1.49)	0.71 (0.32–1.60)		0.84 (0.57–1.25)
**Tumor Size**					
>1 cm (*n* = 362)	110 (30.4)	174 (48.1)	78 (21.6)	394 (54.4)	330 (45.6)
≤1 cm (*n* = 138)	40 (29.0)	77 (55.8)	21 (15.2)	157 (56.9)	119 (43.1)
OR (95% CI) ^§^	1.00	1.22 (0.78–1.91)	0.74 (0.41–1.35)		0.90 (0.68–1.20)
OR (95% CI) ^#^	1.00	1.21 (0.77–1.91)	0.73 (0.39–1.33)		0.89 (0.67–1.19)
**Lymph Node or Distant Metastasis**					
No (*n* = 385)	113 (29.4)	198 (51.4)	74 (19.2)	424 (55.1)	346 (44.9)
Yes (*n* = 115)	37 (22.2)	53 (46.1)	25 (21.7)	127 (55.2)	103 (44.8)
OR (95% CI) ^§^	1.00	0.82 (0.51–1.32)	1.03 (0.57–1.85)		0.99 (0.74–1.34)
OR (95% CI) ^#^	1.00	0.83 (0.51–1.34)	1.06 (0.59–1.91)		1.01 (0.75–1.36)

*n*, number. OR, odds ratio (using the first column of genotypes or alleles as reference). CI, confidence interval. FTC, follicular thyroid carcinoma. PTC, papillary thyroid carcinoma. ^§^, crude (non-adjusted). ^#^, adjusted for sex and age. ^(a)^ codominant model. ^(b)^ log-additive model.

**Table 2:** Analysis of *CYP2R1* rs2060793 genotype and allele frequencies.

	Genotypes, *n* (%)			Alleles, *n* (%)	
	GG	GA	AA	G	A
**All**					
Controls (*n* = 500)	183 (36.6)	256 (51.2)	61 (12.2)	622 (62.2)	378 (37.8)
Patients (*n* = 500)	189 (37.8)	236 (47.2)	75 (15.0)	614 (61.4)	386 (38.6)
OR (95% CI) ^§^	1.00	0.89 (0.68–1.17)	1.19(0.80–1.77)		1.04 (0.86–1.25)
OR (95% CI) ^#^	1.00	0.88 (0.66–1.16)	1.11 (0.74–1.68)		1.01 (0.83–1.22)
**Tumor Type**					
FTC (*n* = 58)	23 (39.7)	23 (39.7)	12 (20.7)	69 (59.5)	47 (40.5)
PTC (*n* = 442)	116 (37.6)	213 (48.2)	63 (14.2)	545 (61.7)	339 (38.3)
OR (95% CI) ^§^	1.00	1.28 (0.70–2.37)	0.73 (0.34–1.55)		0.91 (0.62–1.91)
OR (95% CI) ^#^	1.00	1.32 (0.71–2.46)	0.72 (0.33–1.54)		0.91 (0.61–1.36)
**Tumor Size**					
>1 cm (*n* = 362)	131 (36.2)	172 (47.5)	59 (16.3)	434 (59.9)	290 (40.1)
≤1 cm (*n* = 138)	58 (42.0)	64 (46.4)	16 (11.6)	180 (65.2)	96 (34.8)
OR (95% CI) ^§^	1.00	0.84 (0.55–1.28)	0.61 (0.33–1.15)		0.80 (0.60–1.07)
OR (95% CI) ^#^	1.00	0.83 (0.54–1.27)	0.58 (0.31–1.10)		0.78 (0.58–1.05)
**Lymph Node or Distant Metastasis**					
No (*n* = 385)	135 (35.1)	188 (48.8)	62 (16.1)	458 (59.5)	312 (40.5)
Yes (*n* = 115)	54 (47.0)	48 (41.7)	13 (11.3)	156 (67.8)	74 (32.2)
OR (95% CI) ^§^	1.00	0.64 (0.41–1.00)	0.52 (0.27–1.03) 0.61 (0.40–0.93) *p* = 0.022 ^(a)^		0.70 (0.51–0.95) *p* = 0.022 ^(b)^
OR (95% CI) ^#^	1.00	0.64 (0.41–1.01)	0.54 (0.27-1.07) 0.62 (0.40–0.94) *p* = 0.027 ^(a)^		0.71 (0.51–0.97) *p* = 0.028 ^(b)^

*n*, number. OR, odds ratio (using the first column of genotypes or alleles as a reference). CI, confidence interval. FTC, follicular thyroid carcinoma. PTC, papillary thyroid carcinoma. ^§^, crude (non-adjusted). ^#^, adjusted for sex and age. ^(a)^ dominant model. ^(b)^ log-additive model.

**Table 3:** Analysis of *CYP24A1* rs6013897 genotype and allele frequencies.

	Genotypes, *n* (%)			Alleles, *n* (%)	
	TT	TA	AA	T	A
**All**					
Controls (*n* = 500)	310 (62.1)	171 (34.3)	18 (3.6)	791 (79.3)	207 (20.7)
Patients (*n* = 500)	302 (60.4)	170 (34.0)	28 (5.6)	774 (77.4)	226 (22.6)
OR (95% CI) ^§^	1.00	1.02 (0.78–1.33)	1.06 (0.87–2.95)		1.12 (0.90–1.38)
OR (95% CI) ^#^	1.00	1.03 (0.78–1.35)	1.66 (0.88–3.14)		1.13 (0.91–1.41)
**Tumor Type**					
FTC (*n* = 58)	34 (58.6)	19 (32.8)	5 (8.6)	87 (75.0)	29 (25.0)
PTC (*n* = 442)	268 (60.6)	151 (34.2)	23 (5.2)	687 (77.7)	197 (22.3)
OR (95% CI) ^§^	1.00	1.01 (0.56–1.83)	0.58 (0.21–1.64)		0.86 (0.55–1.35)
OR (95% CI) ^#^	1.00	1.04 (0.57–1.89)	0.56 (0.20–1.57)		0.86 (0.55–1.36)
**Tumor Size**					
>1 cm (*n* = 362)	214 (59.1)	130 (35.9)	18 (5.0)	558 (77.1)	166 (22.9)
≤1 cm (*n* = 138)	88 (63.8)	40 (29.0)	10 (7.2)	216 (78.3)	60 (21.7)
OR (95% CI) ^§^	1.00	0.75 (0.49–1.15)	1.35 (0.60–3.04)		0.94 (0.67–1.30)
OR (95% CI) ^#^	1.00	0.75 (0.49–1.17)	1.39 (0.61–3.15)		0.95 (0.68–1.32)
**Lymph Node or Distant Metastasis**					
No (*n* = 385)	231 (60.0)	130 (33.8)	24 (6.2)	592 (76.9)	178 (23.1)
Yes (*n* = 115)	71 (61.7)	40 (34.8)	4 (3.5)	182 (79.1)	48 (20.9)
OR (95% CI) ^§^	1.00	1.00 (0.64–1.56)	0.54 (0.18–1.62)		0.88 (0.62–1.26)
OR (95% CI) ^#^	1.00	0.99 (0.64–1.55)	0.52 (0.18–1.57)		0.87 (0.61–1.24)

*n*, number. OR, odds ratio (using the first column of genotypes or alleles as a reference). CI, confidence interval. FTC, follicular thyroid carcinoma. PTC, papillary thyroid carcinoma. ^§^, crude (non-adjusted). ^#^, adjusted for sex and age.

## References

[1] Maniakas A., Davies L., Zafereo M.E. (2018). Thyroid Disease Around the World. Otolaryngol. Clin. N. Am..

[2] Landa I., Robledo M. (2011). Association studies in thyroid cancer susceptibility: Are we on the right track?. J. Mol. Endocrinol..

[3] Saenko V.A., Rogounovitch T.I. (2018). Genetic Polymorphism Predisposing to Differentiated Thyroid Cancer: A Review of Major Findings of the Genome-Wide Association Studies. Endocrinol. Metab..

[4] Hu M.J., Zhang Q., Liang L., Wang S.Y., Zheng X.C., Zhou M.M., Yang Y.W., Zhong Q., Huang F. (2018). Association between vitamin D deficiency and risk of thyroid cancer: A case-control study and a meta-analysis. J. Endocrinol. Investig..

[5] Zhao J., Wang H., Zhang Z., Zhou X., Yao J., Zhang R., Liao L., Dong J. (2019). Vitamin D deficiency as a risk factor for thyroid cancer: A meta-analysis of case-control studies. Nutrition.

[6] Campbell M.J., Trump D.L. (2017). Vitamin D Receptor Signaling and Cancer. Endocrinol. Metab. Clin. N. Am..

[7] Clinckspoor I., Verlinden L., Mathieu C., Bouillon R., Verstuyf A., Decallonne B. (2013). Vitamin D in thyroid tumorigenesis and development. Prog. Histochem. Cytochem..

[8] Bouillon R. (2017). Genetic and Racial Differences in the Vitamin D Endocrine System. Endocrinol. Metab. Clin. N. Am..

[9] Bahrami A., Sadeghnia H.R., Tabatabaeizadeh S.A., Bahrami-Taghanaki H., Behboodi N., Esmaeili H., Ferns G.A., Mobarhan M.G., Avan A. (2018). Genetic and epigenetic factors influencing vitamin D status. J. Cell Physiol..

[10] Ahn J., Yu K., Stolzenberg-Solomon R., Simon K.C., McCullough M.L., Gallicchio L., Jacobs E.J., Ascherio A., Helzlsouer K., Jacobs K.B. (2010). Genome-wide association study of circulating vitamin D levels. Hum. Mol. Genet..

[11] Wang T.J., Zhang F., Richards J.B., Kestenbaum B., van Meurs J.B., Berry D., Kiel D.P., Streeten E.A., Ohlsson C., Koller D.L. (2010). Common genetic determinants of vitamin D insufficiency: A genome-wide association study. Lancet.

[12] Raimundo J., Alvelos M.I., Azevedo T., Martins T., Rodrigues F.J., Lemos M.C. (2017). Association of FOXE1 polyalanine repeat region with thyroid cancer is dependent on tumour size. Clin. Endocrinol..

[13] Karczewski K.J., Francioli L.C., Tiao G., Cummings B.B., Alföldi J., Wang Q., Collins R.L., Laricchia K.M., Ganna A., Birnbaum D.P. (2019). Variation across 141,456 human exomes and genomes reveals the spectrum of loss-of-function intolerance across human protein-coding genes. bioRxiv.

[14] Lemos M.C., Regateiro F.J. (1998). N-acetyltransferase genotypes in the Portuguese population. Pharmacogenetics.

[15] Untergasser A., Nijveen H., Rao X., Bisseling T., Geurts R., Leunissen J.A. (2007). Primer3Plus, an enhanced web interface to Primer3. Nucleic Acids Res..

[16] Sole X., Guino E., Valls J., Iniesta R., Moreno V. (2006). SNPStats: A web tool for the analysis of association studies. Bioinformatics.

[17] Dupont W.D., Plummer W.D. (1998). Power and sample size calculations for studies involving linear regression. Control Clin. Trials.

[18] Theodoratou E., Palmer T., Zgaga L., Farrington S.M., McKeigue P., Din F.V., Tenesa A., Davey-Smith G., Dunlop M.G., Campbell H. (2012). Instrumental variable estimation of the causal effect of plasma 25-hydroxy-vitamin D on colorectal cancer risk: A mendelian randomization analysis. PLoS ONE.

[19] Hiraki L.T., Qu C., Hutter C.M., Baron J.A., Berndt S.I., Bezieau S., Brenner H., Caan B.J., Casey G., Chang-Claude J. (2013). Genetic predictors of circulating 25-hydroxyvitamin d and risk of colorectal cancer. Cancer Epidemiol. Biomark. Prev..

[20] Dimitrakopoulou V.I., Tsilidis K.K., Haycock P.C., Dimou N.L., Al-Dabhani K., Martin R.M., Lewis S.J., Gunter M.J., Mondul A., Shui I.M. (2017). Circulating vitamin D concentration and risk of seven cancers: Mendelian randomisation study. BMJ.

[21] Mondul A.M., Shui I.M., Yu K., Travis R.C., Stevens V.L., Campa D., Schumacher F.R., Ziegler R.G., Bueno-de-Mesquita H.B., Berndt S. (2013). Genetic variation in the vitamin d pathway in relation to risk of prostate cancer—Results from the breast and prostate cancer cohort consortium. Cancer Epidemiol. Biomark. Prev..

[22] Sun Y.Q., Brumpton B.M., Bonilla C., Lewis S.J., Burgess S., Skorpen F., Chen Y., Nilsen T.I.L., Romundstad P.R., Mai X.M. (2018). Serum 25-hydroxyvitamin D levels and risk of lung cancer and histologic types: A Mendelian randomisation analysis of the HUNT study. Eur. Respir. J..

[23] Ong J.S., Gharahkhani P., An J., Law M.H., Whiteman D.C., Neale R.E., MacGregor S. (2018). Vitamin D and overall cancer risk and cancer mortality: A Mendelian randomization study. Hum. Mol. Genet..

[24] Penna-Martinez M., Ramos-Lopez E., Stern J., Kahles H., Hinsch N., Hansmann M.L., Selkinski I., Grunwald F., Vorlander C., Bechstein W.O. (2012). Impaired vitamin D activation and association with CYP24A1 haplotypes in differentiated thyroid carcinoma. Thyroid.

[25] Santos M., Azevedo T., Martins T., Rodrigues F.J., Lemos M.C. (2014). Association of RET genetic polymorphisms and haplotypes with papillary thyroid carcinoma in the Portuguese population: A case-control study. PLoS ONE.

[26] Jendrzejewski J., Liyanarachchi S., Nagy R., Senter L., Wakely P.E., Thomas A., Nabhan F., He H., Li W., Sworczak K. (2016). Papillary Thyroid Carcinoma: Association Between Germline DNA Variant Markers and Clinical Parameters. Thyroid.

[27] Anic G.M., Thompson R.C., Nabors L.B., Olson J.J., Browning J.E., Madden M.H., Murtagh F.R., Forsyth P.A., Egan K.M. (2012). An exploratory analysis of common genetic variants in the vitamin D pathway including genome-wide associated variants in relation to glioma risk and outcome. Cancer Causes Control.

[28] Kim D. (2017). The Role of Vitamin D in Thyroid Diseases. Int. J. Mol. Sci..

[29] Prabhu A.V., Luu W., Li D., Sharpe L.J., Brown A.J. (2016). DHCR7: A vital enzyme switch between cholesterol and vitamin D production. Prog. Lipid Res..

[30] Kuzu O.F., Noory M.A., Robertson G.P. (2016). The Role of Cholesterol in Cancer. Cancer Res..

[31] Elkum N., Alkayal F., Noronha F., Ali M.M., Melhem M., Al-Arouj M., Bennakhi A., Behbehani K., Alsmadi O., Abubaker J. (2014). Vitamin D insufficiency in Arabs and South Asians positively associates with polymorphisms in GC and CYP2R1 genes. PLoS ONE.

